# Peripheral levels of circulating progenitor cells correlate with area at risk and microvascular obstruction at cardiac magnetic resonance imaging in patients with ST-elevation myocardial infarction

**DOI:** 10.1186/1532-429X-13-S1-P240

**Published:** 2011-02-02

**Authors:** Italo Porto, Christian Hamilton Craig, Giovanni Luigi De Maria, Antonio Maria Leone, Alessandra Tritarelli, Francesco Burzotta, Claudia Camaioni, Luigi Natale, Luigi Marzio Biasucci, Filippo Crea

**Affiliations:** 1 Catholic University of the Sacred Heart, Rome, Italy

## Background

Circulating progenitor cells (CPC) are bone marrow-derived elements with reparative properties known to be mobilised in the context of acute coronary syndromes. Controversy persists over the determinants of their release in the acute phase of ST-elevation myocardial infarction (STEMI). We used cardiovascular magnetic resonance (CMR) to investigate this issue.

## Methods

Fifteen STEMI patients undergoing successful primary percutaneous coronary intervention (pPCI) were included. Peripheral vein blood samples were drawn at 48 hours after pPCI to measure CPC (CD34+/ KDR+/CD45-). At 2-7 days after pPCI all patients underwent CMR assessment with SSFP, T2-STIR, first-pass perfusion and late gadolinium-enhancement imaging. Quantitative T2 area-at-risk (AAR, % of LV volume), infarct size (IS, % of LV volume), myocardial salvage (MS, calculated as AAR-IS) were calculated. Microvascular obstruction (MVO) was assessed both as perfusion defect score on first-pass imaging (PD, expressed as a sum of a 0 to 3 scoring for each of 16 LV segments) or as MVO score in the late enhancement images.

## Results

CPC levels (expressed as %, number of cells per total number of cytometric events) were more strongly related to extent of AAR (rho=0.72, p=0.002) than to IS (rho=0.5, p=0.06), but did not correlate to MS (rho=0.002, p=0.9). Moreover, CPC were related to PD score (rho=0.54, p=0.03) but not to MVO score (rho=0.3, p=0.2).

## Conclusions

Our data suggest that CPC are mobilized in STEMI patients in response to the total myocardial ischemic insult, as represented by the area-at-risk on T2-STIR. Furthermore, CPC positive correlation with PD suggests that they could be mobilized in attempt to overcome ongoing microvascular dysfunction.

